# Case report: Novel multi-exon homozygous deletion of *ZBTB24* causes immunodeficiency, centromeric instability, and facial anomalies syndrome 2

**DOI:** 10.3389/fimmu.2025.1517417

**Published:** 2025-01-31

**Authors:** Yan Long, Chenghan Wang, Jie Xiao, Yunhua Huang, Xiaoting Ling, Chaoyu Huang, Ying Chen, Jiaqi Luo, Rongheng Tang, Faquan Lin, Yifang Huang

**Affiliations:** ^1^ Department of Clinical Laboratory, The First Affiliated Hospital of Guangxi Medical University, Key Laboratory of Clinical Laboratory Medicine of Guangxi Department of Education, Nanning, Guangxi, China; ^2^ Department of Pediatrics, Rong’an County People’s Hospital, Liuzhou, Guangxi, China

**Keywords:** case report, *ZBTB24*, ICF syndrome 2, nonsense variant, intravenous immunoglobulin

## Abstract

Immunodeficiency, centromeric instability, and facial anomalies syndrome (ICF) is a rare genetic disease characterized by hypogammaglobulinemia, T cell immune deficiency with age, pericentromeric hypomethylation, facial abnormalities, and intellectual disability. This study aimed to investigate the phenotype and immune function of a girl with ICF2, identify her genetic defect, and explore the potential pathogenic mechanisms of the disease. We identified a homologous deletion mutation in this girl, which involves exons 1-5 and part of introns 1 and 6 of the *ZBTB24* gene (NG_029388.1: g.2831_18,995del). This *ZBTB24* variant produces a severely truncated ZBTB24 protein that lacks the BTB, A-T hook and eight zinc fingers. The above changes may lead to abnormal transcriptional function of the ZBTB24 protein. Karyotype analysis showed fragile sites and entire arm deletions were detected on chromosomes 1 and 16 and triradials on chromosome 16. The novel multi-exon deletion of *ZBTB24* causes immunodeficiency, severe pneumonia and centromeric instability in the patient. During the follow-up, the patient’s pneumonia continued to progress despite receiving intravenous immunoglobulin (IVIG) replacement and anti-infective therapy. These results indicated that this novel multi-exon deletion variant of *ZBTB24* may be the genetic etiology of ICF2. The discovery of this novel mutation expands the mutation spectrum of the *ZBTB24* gene and improves our understanding of the molecular mechanisms underlying ICF.

## 1 Introduction

Immunodeficiency, centromeric instability, and facial anomalies syndrome (ICF) is a rare autosomal recessive genetic disease and one of the earliest congenital diseases discovered to be caused by DNA methylation defects (1, 2). To date, only approximately 120 cases of ICF have been reported worldwide (3). ICF is characterized by a significant reduction in immunoglobulin levels that can lead to recurrent infections in respiratory and gastrointestinal systems, facial abnormalities (such as ocular hypertelorism, epicanthic folds, low-set ears, and flat nose) and intellectual disability (4). According to the molecular genetic evidence, ICF can be divided into five subtypes: about 60% of patients carry *DNMT3B* variants, known as ICF syndrome 1 (ICF1, OMIM 242860); nearly 30% of ICF individuals belong to ICF syndrome 2 (ICF2, OMIM 614069), caused by variants in *ZBTB24*; ICF syndrome 3 (ICF3, OMIM 616910) and ICF syndrome 4 (ICF4, OMIM 616911) are respectively associated with variants in *CDCA7* and *HELLS*; Only very few cases of ICF with unknown causative genes are classified as ICF syndrome X (ICFX) (1, 5).

The human *ZBTB24* gene is located on chromosome 6q21 and contains 7 exons. The protein encoded by this gene is a member of the ZBTB family of transcriptional regulators. ZBTB24 is composed of a BTB (broad-complex, tram-track, and bric-a-brac) domain, an A-T hook domain, and eight C2H2-type zinc fingers (6). Previous study found that low expression of *ZBTB24* was significantly associated with decreased yields and impaired differentiation of mature B lymphocytes, resulting in a higher incidence of hypogammaglobulinemia in ICF2 patients (7). Although ICF patients with different subtypes show similar clinical phenotypes, the clinical outcomes can vary depending on the severity and frequency of infection (5, 8, 9). Weemaes et al. found that a majority of the 44 patients with ICF suffered from severe pneumonia, resulting in the mortality rate as high as 34.09%. The age range at death for ICF1 patients was 0.75-19 years, and for ICF2 patients it was 4-13 years (10). The study above highlights the importance of early detection and intervention in cases of ICF due to its rapid progression and unfavorable prognosis. And ICF is often underdiagnosed because of the rarity and complexity of the disease. Therefore, more and more studies are devoted to revealing the immunological and genetic characteristics of ICF to help standardize the diagnosis and treatment of ICF (9, 11, 12). In this study, we continuously monitored the immune status and evolution of lung inflammation in an ICF2 patient and discovered a novel *ZBTB24* variant that includes a homologous deletion of exons 1-5. The findings expand the mutation spectrum of *ZBTB24* and reveal novel molecular defects associated with ICF2.

## 2 Materials and methods

### 2.1 Subjects

A 9-year-old girl diagnosed with ICF2 was enrolled in our study and her examination results and peripheral blood samples were collected. Written informed consent from the legal guardian of the child and informed assent from the child were obtained. This work was conducted in accordance with the guidelines of the Helsinki Declaration and approved by the First Affiliated Hospital of Guangxi Medical University Ethical Review Committee.

### 2.2 Whole-exome sequencing

Genomic DNA was extracted from the patient’s blood sample and fragmented according to the manufacturer’s instructions. The enriched exome library was sequenced on the Illumina HiSeq 2000 platform. After quality filtering the raw data using cutadapt (version 1.16), clean reads were mapped to human reference genome GRCh37/hg19 by the Burrows-Wheeler Aligner (BWA) software. Finally, the variants identified in the patient’s DNA were annotated using ANNOVAR software and the pathogenicity of each variant was assessed using REVEL.

### 2.3 Validation of *ZBTB24* variant

The exon deletion was verified by quantification of the DNA copy number of exons 2-7 using real-time quantitative PCR (qPCR), and the data were normalized to *GAPDH*. Exon 1 with high GC content was amplified using the following PCR system: a total reaction volume of 25 μL containing LA Taq polymerase 1.25 U, 2×GC Buffer I 12.5 μL, dNTP Mixture 400 μM (Takara Bio, Dalian, China), 0.4 μM of each primer and 200 ng of genomic DNA template. Amplification parameters were as follows: pre-denaturation at 94°C for 1 min, denaturation at 94°C for 30 s, and annealing extension at 68°C for 5 min in the first 10 cycles. In the following 20 cycles, the annealing time was increased by 20 s per cycle. A final extension at 68°C for 10 min was performed. The products were separated by agarose gel electrophoresis and subjected to Sanger sequencing.

In order to locate the specific site of the deleted sequence, we designed primers targeting two short fragments (N1 and N2) of intron 1 and four short fragments (C1-C4) of intron 6 and performed qPCR. We selected the forward primer of fragment N1 and the reverse primer of fragment C4 for gap-PCR when only fragments N1 and C4 showed typical amplification curves. The reagents and experimental procedures were the same as those for exon 1 described above. All experiments used genomic DNA from normal individuals as controls. Primer sequences are shown in **Supplementary Table S1**. Primers for *GAPDH* were purchased from Biochuangyi Bio Inc. (Shanghai, China).

### 2.4 Protein mutation analysis

The translated amino acid sequence of this *ZBTB24* variant was analyzed using SnapGene (v.6.0.2). The three-dimensional structural models of wild-type and mutant ZBTB24 proteins were predicted using SWISS-MODEL software (https://swissmodel.expasy.org/) for visualization and comparison.

### 2.5 G-banded karyotype analysis

Firstly, cell culture and phytohemagglutinin (PHA) stimulation to induce cell division were performed. Then, colchicine was added to stop the cells from dividing. After hypotonicity treatment and fixation, Giemsa staining was used. Cytogenetic analysis was performed on 100 metaphase chromosome images using Ikaros karyotyping software with a resolution of 400-550 bands. The karyotypes were described according to ISCN 2020 (13).

## 3 Results

### 3.1 Case information

The patient was a 9-year-old girl who had suffered from recurrent respiratory infections since the age of 3 months, with more than 7 episodes per year. The previous infections had improved with antibacterial treatment. In November 2023, the girl was transferred to our hospital because the recurrent infection progressed to severe pneumonia. Physical examination revealed that the child was 122 cm tall (<-3SD) and weighed 20 kg (<-2SD). She had slightly wider eyes, a flat nose, and low-set ears. It was also learned that the patient had difficulty understanding complex courses and was considered to have a mild intellectual disability according to a total score of 72 on the Chinese-Wechsler Intelligence Scale for Children (C-WISC). These results suggest that the girl has growth and cognitive delays, with motor and speech development similar to that of her peers.

**Figure 1:**
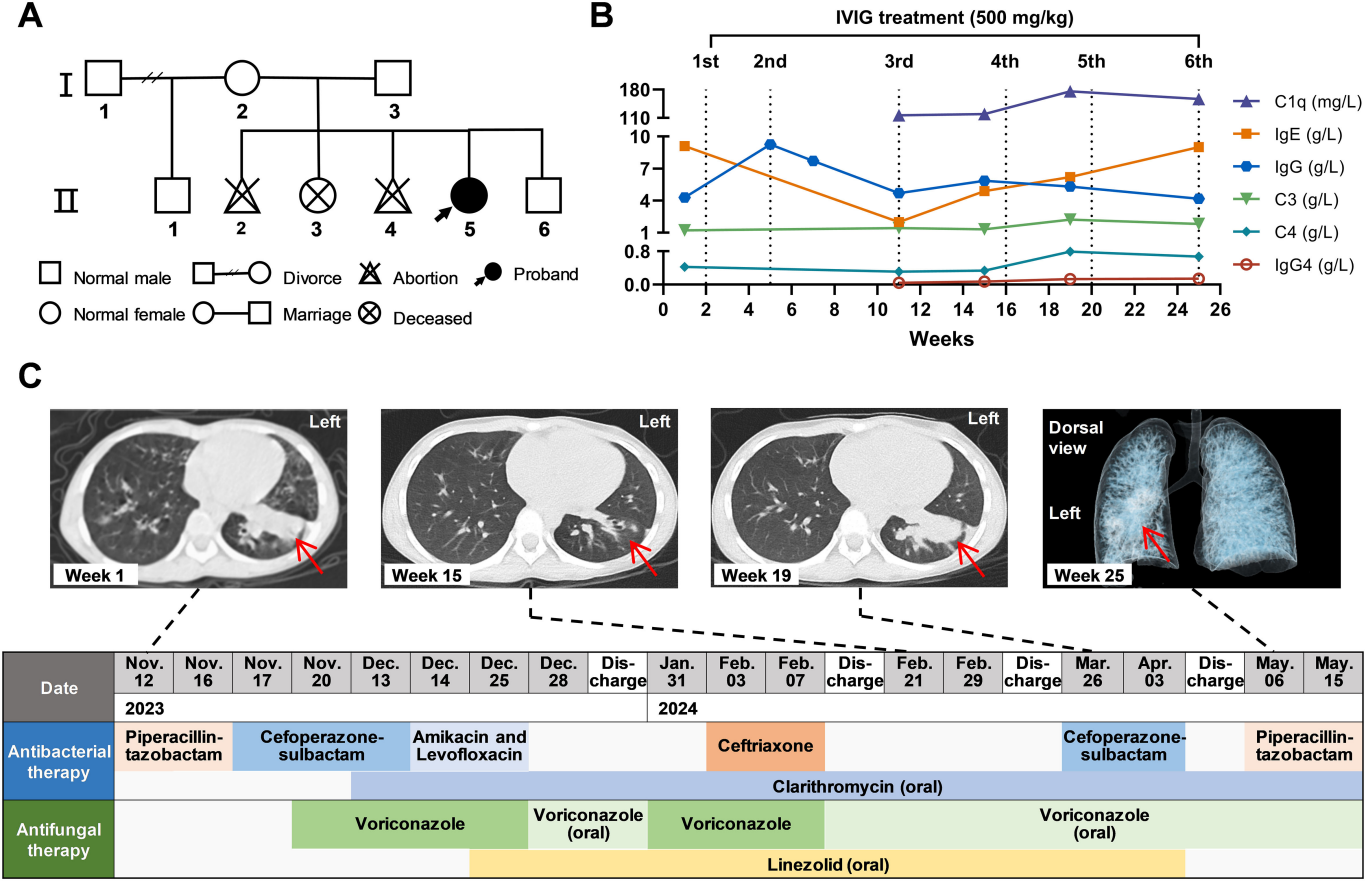
Clinical characteristics of the ICF2 patient. **(A)** Family pedigree of this proband. **(B)** The levels of various immunoglobulins and complement during follow-up. The number and dose of IVIG treatment are indicated above the line graphs. **(C)** Timeline of antibacterial and antifungal treatment. Antibiotics were given intravenously except for those marked “oral”. Above the timeline are chest CT at weeks 1, 15, and 19 and three-dimensional reconstruction of the bronchi at week 25. The red arrows point to the lung lesions.

Further family investigation (**Figure 1A**) revealed that the parents of this proband (II-5) had no immunodeficiency and were not consanguineous. However, the proband’s sister (II-3) had growth retardation and died of unknown causes when she was less than 10 years old. During the fourth spontaneous conception, her mother discovered that the fetus (II-4) had facial abnormalities by antenatal examination and underwent an induced abortion. After the birth of the proband, her mother gave birth to a healthy boy (II-6), who is now 7 years old.

### 3.2 Immunologic monitoring and replacement therapy

The immunological assessment of the patient before intravenous immunoglobulin (IVIG) treatment indicated immunoglobulin (Ig) G at 4.29 g/L (**Figure 1B**), IgA at 0.47 g/L, and low levels of IgM at 0.01 g/L. The anti-tetanus toxoid IgG content was 0.09 IU/mL, indicating a poor antibody response to the tetanus toxoid vaccine. Lymphocyte subpopulation analysis in the first week showed that the proportion of total T cells was normal (72.03%), the CD4+/CD8+ ratio was 1.35, the proportion of B cells was increased (25.37%), and the proportion of NK cells was significantly low (1.07%). The proportion of T cells increased to 83.90% (4692 cells/μL), and the proportion of B cells decreased (12.57%; 725 cells/μL) at week 11. The results of complement C3, complement C4, autoantibody testing, and allergen testing were essentially normal. The patient then received IVIG replacement treatment at a dose of 500 mg/kg each time at weeks 2, 5, 11, 16, 20, and 25, respectively. After the first IVIG treatment, the IgG level could rise to 9.25 g/L, but the IgG levels were lower than the normal ranges and fluctuated between 4.17-5.82 g/L since the third treatment. Specific values of laboratory tests are shown in **Supplementary Table S2**.

### 3.3 Manifestations and progression of infection

Blood tests on admission revealed an increased white blood cell (WBC) count of 15.79×10^9^/L, aspartate aminotransferase (AST) level of 84 U/L, and alanine aminotransferase (ALT) level of 99 U/L (**Supplementary Table S2**). Computed tomography (CT) of the chest showed patchy and linear increased density shadows in the lower lingual segment of the upper lobe and the anterior inner basal segment of the lower lobe in the left lung (**Figure 1C**; **Supplementary Figure S1**). Unexpectedly, multiple Gram and acid-fast stains, bacterial and fungal cultures of sputum and bronchoalveolar lavage fluid (BALF) were negative. Tuberculin skin test and interferon-gamma release assay (IGRA) yielded negative results. The fifth G (1,3-β-D-glucan) test and GM (galactomannan) test of BALF were positive, but the previous four tests were negative. Moreover, metagenomic next-generation sequencing (mNGS) of BALF did not detect highly pathogenic microorganisms.

The patient’s infection symptoms improved, and the CT scan at week 15 showed lung inflammation subsided significantly after receiving IVIG, antibacterial, and antifungal treatment (**Figure 1C**). Antituberculosis therapy was not given because there was no definitive evidence of mycobacterium tuberculosis infection. However, this patient developed a persistent high fever (39°C ± 0.5°C) at week 19, and the CT scan at that time revealed severe lung inflammation compared with that at week 15. The reconstructed three-dimensional bronchial model at week 25 suggested retention of secretions in the left lower lobe bronchus.

### 3.4 Identification of novel genetic variant

**Figure 2:**
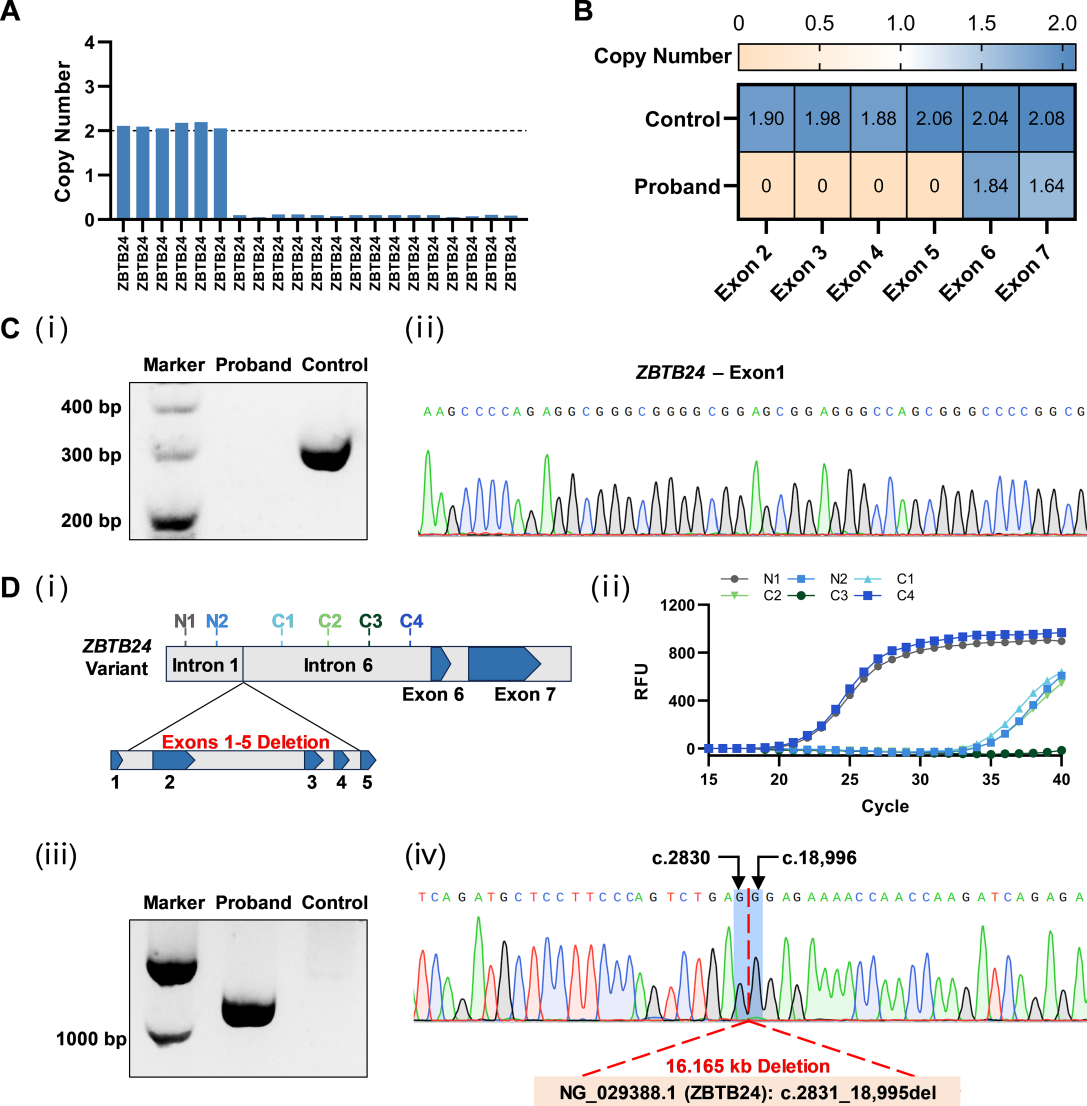
Identification of the novel *ZBTB24* variant. **(A)** Copy number loss of multi-exons of *ZBTB24* was detected by WES. **(B)** The deletion of exons 2-5 in *ZBTB24* was confirmed by qPCR. **(C)** i: Agarose gel electrophoresis showed the expected band of about 300 bp in the control group while no band in the patient’s lane. ii: The PCR product of the control group was clearly found to contain exon 1 of *ZBTB24* by Sanger sequencing. **(D)** i: Two pairs of primers were designed in intron 1 (N1 and N2), and four pairs of primers were designed in intron 6 (C1-C4). ii: The results of qPCR using these six primer pairs indicated that only fragments N1 and C4 were amplified normally. iii: Agarose gel electrophoresis showed that the patient had a DNA fragment (about 1000 bp) containing the deleted sequence information in gap-PCR, while the control group was negative. iv: Sanger sequencing results for the breakpoint position analysis using gap-PCR product.

WES was performed and identified a novel variant of homologous deletions of exons 2, 3, 4, and 5 on *ZBTB24* gene (**Figure 2A**). Subsequently, qPCR was used to amplify the DNA fragment of exons 2-7 to verify the deletion of genomic DNA. The results of qPCR confirmed the homozygous deletion of exons 2-5, while exons 6 and 7 were not deleted (**Figure 2B**; **Supplementary Figure S2**). LA Taq polymerase with GC Buffer was employed to amplify exon 1, a GC-rich DNA region, to prevent missed detection from insufficient sequencing depth of WES. Agarose gel electrophoresis and Sanger sequencing results (**Figure 2C**) showed that the control group of the normal individual had the target band whose DNA sequence was consistent with the exon 1 sequence, while no band can be detected in the patient’s lane. These data complemented the WES results and suggested the deletion of exons 1-5 on *ZBTB24*.

Since the 1st and 6th introns of *ZBTB24* are approximately 12 kb in length and the breakpoint positions are unknown, it is difficult to directly determine the deletion variant by gap-PCR. Therefore, as shown in **Figure 2D**, we first screened the expression of six regions using six pairs of qPCR primers and revealed that only the fragment N1 of intron 1 and the fragment C4 of intron 6 were expressed at normal levels. Subsequently, a gap-PCR product containing the deletion variant information was successfully amplified using the forward primer of fragment N1 and the reverse primer of fragment C4. After Sanger sequencing verification, we found that this *ZBTB24* variant had a deletion length of 16.165 kb and was described as NG_029388.1: g.2831_18,995del. These results further confirmed the deletion of the whole of exons 1-5 on *ZBTB24* of the patient. There is no record of this variant in current public databases or literature. The variant is classified as likely pathogenic according to the ACMG guidelines (14).

### 3.5 Characterization of mutant ZBTB24 protein

**Figure 3:**
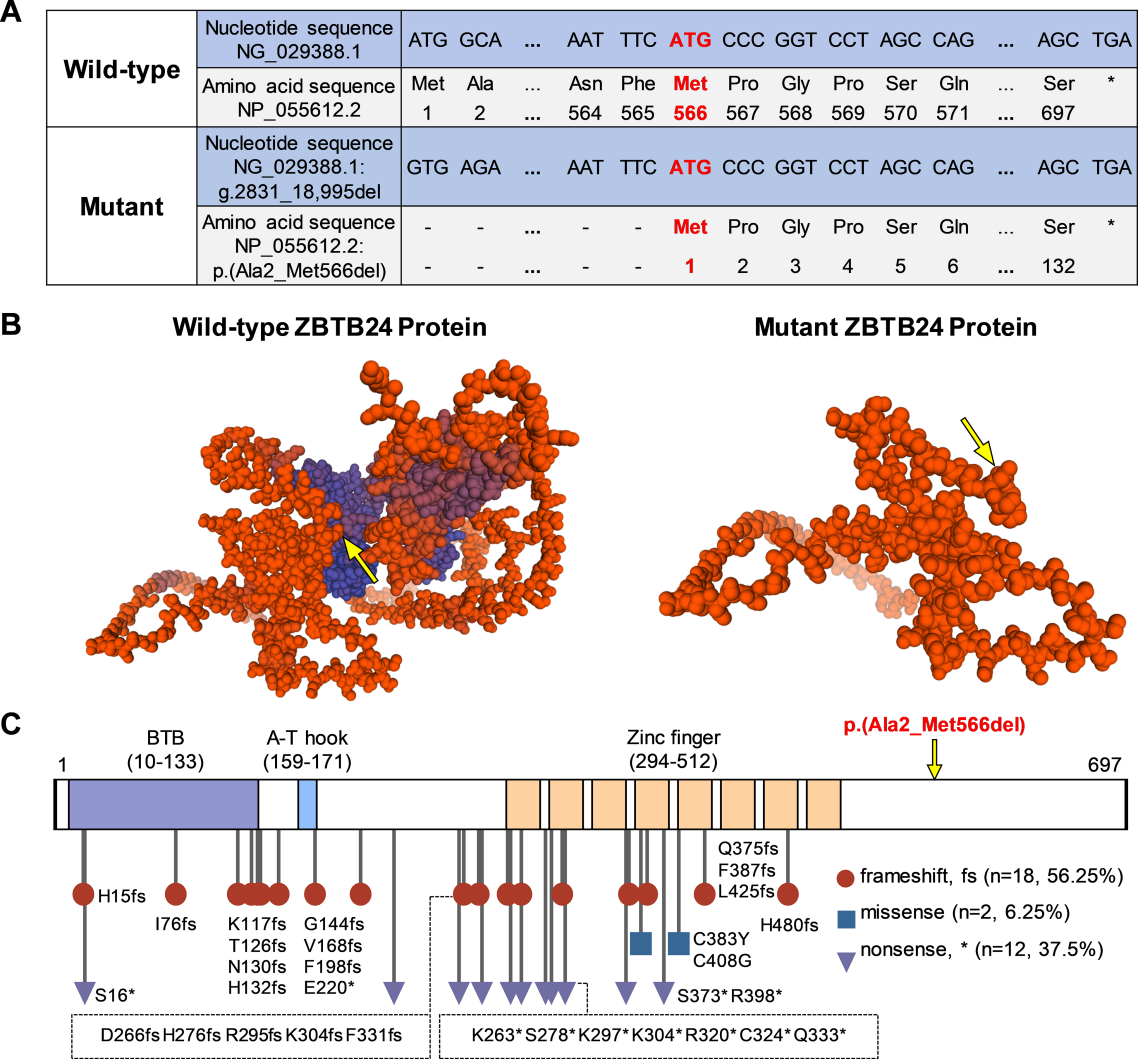
Effects of *ZBTB24* variants on protein structure. **(A)** Through sequence comparison, we found that the start codon (ATG) of this *ZBTB24* variant is located in exon 7, corresponding to the 566th amino acid (Met) of the wild-type, and the stop codon remains unchanged. **(B)** The three-dimensional predicted models of wild-type and mutant ZBTB24 proteins. The yellow arrow points to the original amino acid 566 (Met). **(C)** The amino acid sequence and known variant sites of *ZBTB24*. The symbol "*" marks the stop codon.

The wild-type ZBTB24 protein (NP_055612.2) has a total of 697 amino acids, which include the BTB, A-T hook, and eight zinc fingers. In our study, we found that this multi-exon homozygous deletion variant in *ZBTB24* results in the deletion of the original start codon (ATG) located in exon 2 and the shutoff of subsequent series of amino acids synthesis. The new translation start point is likely to be located at the first ATG codon (g.21,989_21,991) in exon 7, encoding the 566th amino acid in the original sequence (**Figure 3A**). The mutant ZBTB24 protein showed a severe truncation with only 132 amino acids corresponding to the disordered region and was recorded as p.(Ala2_Met566del) (**Figure 3B**). Hence, this variant we report causes a transcriptional defect in the ZBTB24 protein because it lacks the necessary structure made up of a BTB, an A-T hook, and eight zinc fingers.

*ZBTB24* variants in the ClinVar database and previous studies (4, 5, 9, 10, 15, 16) were summarized (**Figure 3C**; **Supplementary Table S3**). Among the 32 *ZBTB24* variants, including the present study, frameshift variants accounted for 56.25%, nonsense variants accounted for 37.5%, and only 2 cases were missense variants. It could be observed that most of the known variants were located in the region of three major functional domains, and there were no obvious mutational hotspots. A review of known *ZBTB24* variant cases revealed that different *ZBTB24* variants may present similar phenotypes, such as hypogammaglobulinemia, centromeric instability, facial anomalies, motor development delay, and intellectual disability (**Supplementary Table S4**).

### 3.6 Abnormal chromosome configuration

**Figure 4:**
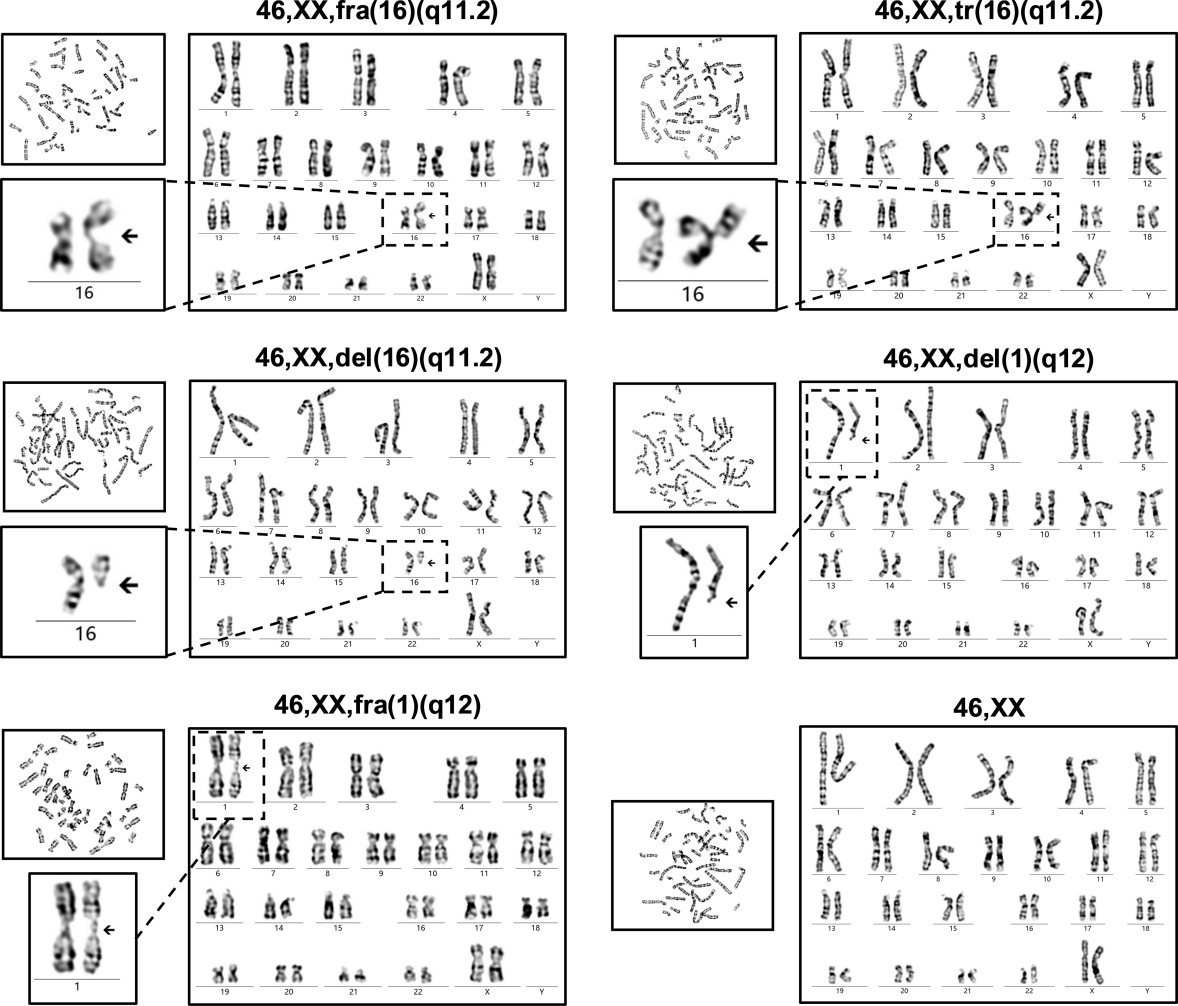
Chromosome karyotype of the ICF2 patient. The chromosome karyotype is described above each set of images. The patient had the characteristic karyotypes associated with DNA hypomethylation in cases with ICF, including fragile sites (fra), whole-arm deletions (del), and triradials (tr).

It was found that fragile site (fra), triradial (tr), and deletion (del) detected on chromosome 16q11.2 in multiple metaphases. The deletion and fragile site were also presented on chromosome 1q12 (**Figure 4**). The patient’s karyotype was described as 46,XX,fra(16)(q11.2)[14]/46,XX,tr(16)(q11.2)[6]/46,XX,del(16)(q11.2)[5]/46,XX,del(1)(q12)[4]/46,XX,fra(1)(q12)[3]/46,XX[68], according to ISCN 2020 (13).

## 4 Discussion

Immunodeficiency, centromeric instability, and facial anomalies syndrome (ICF) is a rare genetic defect that is inherited in an autosomal recessive manner, and the pathogenic genes include *DNMT3B*, *ZBTB24*, *CDCA7*, and *HELLS* (1). Recurrent infections caused by hypogammaglobulinemia are the most prominent clinical symptoms of ICF that prompt these patients to seek medical attention (10). The known cases of ICF were diagnosed based on their symptoms, karyotyping, immune assessment, and genome sequencing results (4). In this study, we initially identified a variant of exons 2-5 deletion of *ZBTB24* associated with ICF2 in a 9-year-old girl with severe pneumonia by WES. During validation, we found that exon 1 was also deleted using PCR targeting GC-rich DNA fragments. Further gap-PCR and Sanger sequencing revealed the exact *ZBTB24* mutation site (NG_029388.1: g.2831_18,995del). Multiple exon deletions of *ZBTB24* are the copy number variations (CNVs) at the exon level with a deletion length of about 16.165 kb. As far as we know, this *ZBTB24* variant is a novel variant that has yet to be documented in public databases.

It is noteworthy that ICF is also one of the first identified diseases with DNA methylation defect (2). DNA methylation is ubiquitous in mammals and maintains genome stability by inhibiting the expression and translocation of genomic transposable elements (17). Unfortunately, ICF patients have widespread hypomethylation, primarily affecting the pericentromeric regions of chromosomes 1, 9, and 16, which are typical targets for hypomethylation of highly repetitive satellite DNA sequences that do not encode proteins (12, 18). Chromosome structural abnormalities can be observed in metaphase images of lymphocytes after stimulation with phytohemagglutinin (PHA) *in vitro* (19). Different chromosomal structural abnormalities were found in our studied case including fragile sites, whole-arm deletions, and triradials. These unusual karyotypes are typical manifestations of DNA hypomethylation and are considered reliable evidence for the diagnosis of ICF (12, 20).

Recent studies have shown that the ZBTB24 protein is a ubiquitously expressed nuclear protein and belongs to the transcription factor family, which is consist of a BTB region, an A-T hook domain, and eight tandem C2H2 zinc fingers (6, 21). The BTB mediates homodimerization, heterodimerization and interactions with other transcriptional coregulators (6). The A-T hook is a functional domain that preferentially binds to short AT-rich DNA fragments (22). The majority of nonsense mutations and frameshift mutations of *ZBTB24* caused abnormalities recorded in the ClinVar database are located in the zinc finger region, indicating that zinc fingers may be the crucial structure for maintaining ZBTB24 protein function. It has been demonstrated that zinc fingers are the structural basis for the ZBTB24 protein to specifically recognize and bind to target DNA and exert its transcriptional regulatory functions (6). The multi-exon homozygous deletion variant of *ZBTB24* that we report here results in the absence of the original start codon, and the shutoff synthesis of subsequent series of amino acids. The severely truncated ZBTB24 protein has only 132 disordered amino acids, which is likely to lead to the loss of its physiological function as a transcription factor.

The functional deficit of the ZBTB24 protein closely correlates with the occurrence and development of immunodeficiency. In general, *ZBTB24* is highly expressed in primary B cells, and downregulation of *ZBTB24* will hinder B cell cycle progression and reduce its proliferation (23). Low expression of *ZBTB24* impedes B lymphocyte differentiation, leading to a significant decrease in immunoglobulin secretion and thus result in primary immunodeficiency (7). The T cell defects also observed in certain ICF2 patients may be due to the mechanism that the deficiency of ZBTB24 protein inhibits T cell proliferation through the CDCA7/TRAIL receptor axis (24). Moreover, the ZBTB24 protein affects multiple cellular pathways as a transcriptional factor, including amino acid metabolism, oxidative stress response, telomere function, apoptosis, and differentiation (21). Consequently, ICF2 patients develop immunodeficiency and are often accompanied by other systemic diseases such as intellectual disability, slow growth and development, facial abnormalities, inflammatory bowel disease (5), autoimmune (25), atrial septal defect (4), and brain cysts (26). Nevertheless, the type and extent of the structural defect in the ZBTB24 protein do not completely correspond to the severity of the immunodeficiency and the complexity of the symptoms (15, 25, 27). At present, it is still difficult to conclude mutation hotspots in *ZBTB24* and genotype-phenotype associations in ICF2, probably due to the limited number of cases in ICF2.

The ICF2 patient we report can be classified as common variable immunodeficiency (CVID) because of recurrent infections, decreased IgG, IgA, and IgM, no secondary causes, a poor response to vaccine, and no profound T cell deficiency (28). The combination approach of intravenous immunoglobulin (IVIG) replacement and anti‐infective is considered to be the first-line treatment option for this immunodeficiency (4, 10, 12). The patient’s parents and physician indicated that the patient’s condition had improved and the frequency of infections had decreased during the early stages of treatment. Regrettably, the patient’s long-term prognosis is unfavorable as primary immunodeficiency cannot be cured with medication. Hematopoietic stem cell transplantation (HSCT) is a radical curative regimen for correcting immunodeficiency in patients with ICF, especially those who have sustained severe infections in infancy or persistent infections that are insufficient with drug treatment (29). There are about 10 cases of ICF children whose life expectancy has been extended through successful HSCT treatment worldwide till now (30, 31).

In summary, we identified a novel pathogenic variant (exons 1-5 del) of *ZBTB24* that is highly correlated with ICF2. A review of previous literature related to *ZBTB24* variants provides a deeper understanding of the underlying mechanisms of ICF and helps to clarify the association between ICF genotype and phenotype.

## Data Availability

The raw data supporting the conclusions of this article will be made available by the authors, without undue reservation.
