# Supplementary material for: Case report: Novel multi-exon homozygous deletion of ZBTB24 causes immunodeficiency, centromeric instability, and facial anomalies syndrome 2
Source: Front Immunol. 2025 Jan 31;16:1517417. doi: 10.3389/fimmu.2025.1517417 (PMC11825828; doi:10.3389/fimmu.2025.1517417)
Supplement: Supplementary file 1 [file DataSheet1.pdf]

CARE
case report guidelines

CARE Checklist of information to include when writing a case report

(cc) BY-NC-ND

Topic	Item	Checklist item description	Reported on Line
**Title**	**1**	The diagnosis or intervention of primary focus followed by the words "case report".	Page 1, Line 1-2
**Key Words**	**2**	2 to 5 key words that identify diagnoses or interventions in this case report, including "case report". .	Page 2, Line 37-38
**Abstract (no references)**	**3a**	Introduction: What is unique about this case and what does it add to the scientific literature?.	Page 2, Line 21-25
	**3b**	Main symptoms and/or important clinical findings	Page 2, Line 25-30
	**3c**	The main diagnoses, therapeutic interventions, and outcomes.	Page 2, Line 30-33
	**3d**	Conclusion—What is the main "take-away" lesson(s) from this case?	Page 2, Line 33-36
**Introduction**	**4**	One or two paragraphs summarizing why this case is unique (**may include references**)	Page 3-4, Line 41-70
**Patient Information**	**5a**	De-identified patient specific information	Page 4, Line 73-74
	**5b**	Primary concerns and symptoms of the patient.	Page 6, Line 116-118
	**5c**	Medical, family, and psycho-social history including relevant genetic information	Page 6-7, Line 118-125
	**5d**	Relevant past interventions with outcomes	Page 6, Line 116-117
**Clinical Findings**	**6**	Describe significant physical examination (PE) and important clinical findings.	Page 6-10, Line 117-194
**Timeline**	**7**	Historical and current information from this episode of care organized as a timeline	Page 7-8, Line 127-147
**Diagnostic Assessment**	**8a**	Diagnostic testing (such as PE, laboratory testing, imaging, surveys).	Page 7-10, Line 124-194
	**8b**	Diagnostic challenges (such as access to testing, financial, or cultural)	Page 8-10, Line 149-194
	**8c**	Diagnosis (including other diagnoses considered)	Page 4, Line 73
	**8d**	Prognosis (such as staging in oncology) where applicable	Page 8, Line 146-147
**Therapeutic Intervention**	**9a**	Types of therapeutic intervention (such as pharmacologic, surgical, preventive, self-care)	Page 7, Line 132-134
	**9b**	Administration of therapeutic intervention (such as dosage, strength, duration)	Page 7, Line 132-134
	**9c**	Changes in therapeutic intervention (with rationale)	N/A, the patient did not undergo further hematopoietic stem cell transplantation.

**Follow-up and Outcomes**	**10a**	Clinician and patient-assessed outcomes (if available)	Page 7, Line 134-136<br>Page 8, Line 146-147
	**10b**	Important follow-up diagnostic and other test results	Page 7, Line 132-134
	**10c**	Intervention adherence and tolerability (How was this assessed?)	N/A, the patient's subsequent diagnosis and testing remained the same as before.
	**10d**	Adverse and unanticipated events	Page 13, Line 249-250
**Discussion**	**11a**	A scientific discussion of the strengths AND limitations associated with this case report	Page 10-11, Line 201-216
	**11b**	Discussion of the relevant medical literature **with references**.	Page 11-13, Line 208-254
	**11c**	The scientific rationale for any conclusions (including assessment of possible causes)	Page 10-13, Line 196-254
	**11d**	The primary "take-away" lessons of this case report (without references) in a one paragraph conclusion	Page 13, Line 252-258
**Patient Perspective**	**12**	The patient should share their perspective in one to two paragraphs on the treatment(s) they received	N/A, the patient received only conservative medical treatment.
**Informed Consent**	**13**	Did the patient give informed consent? Please provide if requested	Yes
