# Supplementary material for: Case report: Novel multi-exon homozygous deletion of ZBTB24 causes immunodeficiency, centromeric instability, and facial anomalies syndrome 2
Source: Front Immunol. 2025 Jan 31;16:1517417. doi: 10.3389/fimmu.2025.1517417 (PMC11825828; doi:10.3389/fimmu.2025.1517417)
Supplement: Supplementary file 2 [file DataSheet2.pdf]

Supplementary Material

1 Supplementary Tables

Supplementary Table S1 PCR primer sequences.

Gene	Forward 5'-3'	Reverse 5'-3'
Exon 1	CGCCCCTCACCTCCCTTC	GGCAGACCTACCGCTCAG
Exon 2	TCAGACGCTCACAGTGACAC	ACCAAAGGTGTCTGCAACCA
Exon 3	CAGGGGAGCGACCTTTCAAA	CACCCTGGGGAATGGGAATC
Exon 4	GGCAGGACAGAAGTCTTTTACC	ACCCTATGTGAACGATATGTGCT
Exon 5	AAAAGGCAGGGAGAGCATGT	GCAGGGCACTCAGAGAACTT
Exon 6	AGAGTTCTCTTCAGACCCACA	ACTGCACACAAAATTCTGCAA
Exon 7	CCTTTCTCCTGCCCTGAGTG	TTTCCTGCTCTCCCGAGGTA
Fragment-N1	TTGCTCCTGTGCTACCAACC	CCCACCTAGGCCTTCCAAAG
Fragment-N2	CAACCCTCGTAGCAGCAAGA	GCCACACATGCTCCTTCTCT
Fragment-C1	TGGTGTTGTGAGTGCGTTGA	CCATCCAAGATGCCACCTCA
Fragment-C2	CCAGTGAGGGAGGTGAACAC	TGTGGGCAACAGAAAGGTGT
Fragment-C3	CCCAGGCTTCTTTCCAGTCT	GGTCTTCCCGCTTAGTGGTT
Fragment-C4	GTGTGCCACTCCCATGAAGT	GCACTACAAACCTTGCCACG

Supplementary Table S2 Laboratory findings of the patient with ICF2.

Examination items	Week 1	Week 5	Week 7	Week 11	Week 15	Week 19	Week 20	Week 25	Reference intervals
IgG (g/L)	4.29	9.25	7.71	4.70	5.85	5.32		4.17	7.00-16.00
IgA (g/L)	0.47	0.29	0.13	0.05	0.04	0.03		0.01	0.70-4.00
IgM (g/L)	0.01	0.11	0.03	0.02	0.05	0.05		0.14	0.40-2.30
IgE (g/L)	9.1			2.0	4.9	6.2		9.0	<200.0
C1q (mg/L)				116.5	119.6	175.9		156.6	157.0-237.0
C3 (g/L)	1.22			1.43	1.31	2.22		1.81	0.70-1.40
C4 (g/L)	0.43			0.31	0.34	0.80		0.67	0.10-0.40
IgG4 (g/L)				0.04	0.07	0.13		0.14	0-2.00
WBC(10^9/L)	15.79	8.80	9.29	9.54	8.71	10.47	8.37	13.87	5.00-12.00
PCT (ng/mL)	0.04	0.04	0.06	0.06	0.05	0.25	0.08	0.18	0-0.05
AST (U/L)	84	78	37	31	29	178	21	29	13-40
ALT (U/L)	99	65	31	20	19	83	39	40	7-45
**Lymphocytes (%)**									
Total T cells	72.03			83.90					62.60-76.80
CD4+ T cells	40.82			41.80					30.00-46.00
CD8+ T cells	30.30			38.10					19.20-33.60
CD4+/CD8+	1.35			1.10					0.95-2.13
Double positive T cells (CD3+CD4-CD8-)	1.26			0.03					
Double negative T cells (CD3+CD4-CD8-)	2.17			4.03					
CD19+ B cells	25.37			12.57					8.50-14.50
Natural Killer Cells	1.07			3.04					9.50-23.50

Supplementary Table 2 (Continued)

Examination items	Week 1	Week 5	Week 7	Week 11	Week 15	Week 19	Week 20	Week 25	Reference intervals
**Absolute lymphocyte count (cells/µL)**									
Total T cells	4042			4692					1185-1901
CD4+ T cells	2299			2336					561-1137
CD8+ T cells	1706			2129					220-1030
CD19+ B cells	1457			725					180-324
Natural Killer Cells	61			176					200-567

IgG: immunoglobulin G; IgA: immunoglobulin A; IgM: immunoglobulin M; IgE: immunoglobulin E; C1q: complement C1q; C3: complement C3; C4: complement C4; IgG4: immunoglobulin G4; WBC: white blood count; PCT: procalcitonin; AST: aspartate aminotransferase; ALT: alanine aminotransferase.

Supplementary Table S3 Known pathogenic *ZBTB24* variants and protein alterations.

ID	Variation	Type	Consequence
2866507	NM_014797.3(ZBTB24):c.44_50del (p.His15fs)	Deletion	frameshift variant
598541	NM_014797.3(ZBTB24):c.47C>G (p.Ser16*)	SNV	nonsense variant
2863511	NM_014797.3(ZBTB24):c.226_227del (p.Ile76fs)	Deletion	frameshift variant
2880060	NM_014797.3(ZBTB24):c.350_351del (p.Lys117fs)	Deletion	frameshift variant
2703195	NM_014797.3(ZBTB24):c.377_378del (p.Thr126fs)	Microsatellite	frameshift variant
2028359	NM_014797.3(ZBTB24):c.389del (p.Asn130fs)	Deletion	frameshift variant
40181	NM_014797.3(ZBTB24):c.396_397del (p.His132fs)	Deletion	frameshift variant
2865191	NM_014797.3(ZBTB24):c.431del (p.Gly144fs)	Deletion	frameshift variant
1526123	NM_014797.3(ZBTB24):c.501dup (p.Val168fs)	Duplication	frameshift variant
2956344	NM_014797.3(ZBTB24):c.593_594del (p.Phe198fs)	Deletion	frameshift variant
2879709	NM_014797.3(ZBTB24):c.658G>T (p.Glu220*)	SNV	nonsense variant
2734924	NM_014797.3(ZBTB24):c.787A>T (p.Lys263*)	SNV	nonsense variant
2915423	NM_014797.3(ZBTB24):c.795dup (p.Asp266fs)	Duplication	frameshift variant
2870778	NM_014797.3(ZBTB24):c.825_844del (p.His276fs)	Deletion	frameshift variant
31095	NM_014797.3(ZBTB24):c.833C>G (p.Ser278*)	SNV	nonsense variant
2809786	NM_014797.3(ZBTB24):c.868_875dup (p.Arg295fs)	Duplication	frameshift variant
951999	NM_014797.3(ZBTB24):c.888dup (p.Lys297*)	Duplication	nonsense variant
1074302	NM_014797.3(ZBTB24):c.909dup (p.Lys304*)	Duplication	nonsense variant
2840471	NM_014797.3(ZBTB24):c.911_914del (p.Lys304fs)	Deletion	frameshift variant
31093	NM_014797.3(ZBTB24):c.958C>T (p.Arg320*)	SNV	nonsense variant
2826131	NM_014797.3(ZBTB24):c.971_972delinsAA (p.Cys324*)	Indel	nonsense variant
2793751	NM_014797.3(ZBTB24):c.993del (p.Phe331fs)	Deletion	frameshift variant
3004627	NM_014797.3(ZBTB24):c.997C>T (p.Gln333*)	SNV	nonsense variant
1353720	NM_014797.3(ZBTB24):c.1118C>G (p.Ser373*)	SNV	nonsense variant
2750593	NM_014797.3(ZBTB24):c.1125_1135del (p.Gln375fs)	Deletion	frameshift variant
2725726	NM_014797.3(ZBTB24):c.1161del (p.Phe387fs)	Deletion	frameshift variant

Supplementary Table S3 (Continued)

ID	Variation	Type	Consequence
650017	NM_014797.3(ZBTB24):c.1192C>T (p.Arg398*)	SNV	nonsense variant
31096	NM_014797.3(ZBTB24):c.1222T>G (p.Cys408Gly)	SNV	missense variant
2841373	NM_014797.3(ZBTB24):c.1272_1281del (p.Leu425fs)	Deletion	frameshift variant
2071437	NM_014797.3(ZBTB24):c.1439dup (p.His480fs)	Duplication	frameshift variant

The information in the above table is recorded in the ClinVar database. The included *ZBTB24* variants are classified as "Pathogenic" by this database. SNV: single nucleotide variant.

Supplementary Table S4 Clinical and genetical features of reported ICF2 patients.

Patient ID	Gender	Status	Consanguinity	Main symptoms	Centromeric instability	Facial anomalies	Growth and intellectual development	Variation	Type	Reference
P1	F	9 years old	No	hypogammaglobulinemia, recurrent pneumonia	Yes	Yes	motor development delay, intellectual disability	g.2831_18,995del p.(Ala2_Met566del)	nonsense	present study
P2	F	died at age 13	Yes	agammaglobulinemia, pneumonia	Yes	Yes	motor development delay, intellectual disability	c.47C>G (p.S16*)	nonsense	[4]
P3	M	6 years old	Yes	agammaglobulinemia	Yes	Yes	intellectual disability	c.759C>G (p.T253*)	nonsense	[4, 10]
P4	F	16 years old	No	hypogammaglobulinemia, recurrent infections and secondary hearing impairment	Yes	Yes	motor development delay, intellectual disability	c.787A>T (p.K263*) and c.980_981del (p.C327fs)	nonsense and frameshift	[15]
P5	F	died at age 41	Yes	agammaglobulinemia, sepsis	Yes	Yes	motor development delay, intellectual disability	c.958C>T (p.R320*)	nonsense	[15]
P6	F	12 years old	No	hypogammaglobulinemia, recurrent pneumonia	Yes	Yes	intellectual disability	c.1369C>T (p.R457*)	nonsense	[9]
P7	M	died at age 4	No	agammaglobulinemia	Yes	Yes	intellectual disability	c.833C>G (p.S278*) and c.1222T>G (p.C408G)	nonsense and missense	[4]
P8	M	13 years old	No	hypogammaglobulinemia	Yes	Yes	motor development delay, intellectual disability	c.396_397del (p.H132fs)	frameshift	[16]
P9	M	13 years old	Yes	agammaglobulinemia	Yes	Yes	intellectual disability	c.501dup (p.V168fs)	frameshift	[4]
P10	M	4 years old	Yes	agammaglobulinemia	Yes	Yes	motor development delay, intellectual disability	c.917delA (p.N306fs)	frameshift	[4]
P11	M	28 years old	No	hypogammaglobulinemia, recurrent pneumonia	NA	Yes	normal	c.1108_1109 insA (p.S370fs)	frameshift	[9]
P12	M	17 months old	Yes	agammaglobulinemia, recurrent respiratory and gastrointestinal infections	Yes	Yes	motor development delay, intellectual disability	c.1492_1493del (p.Q498fs)	frameshift	[5]
P13	M	died at age 7	No	hypogammaglobulinemia, recurrent pneumonia	Yes	Yes	motor development delay, intellectual normal	c.1148G>A (p.C383Y)	missense	[15]

F: female; M: male; NA: not applicable.

2 Supplementary Figures

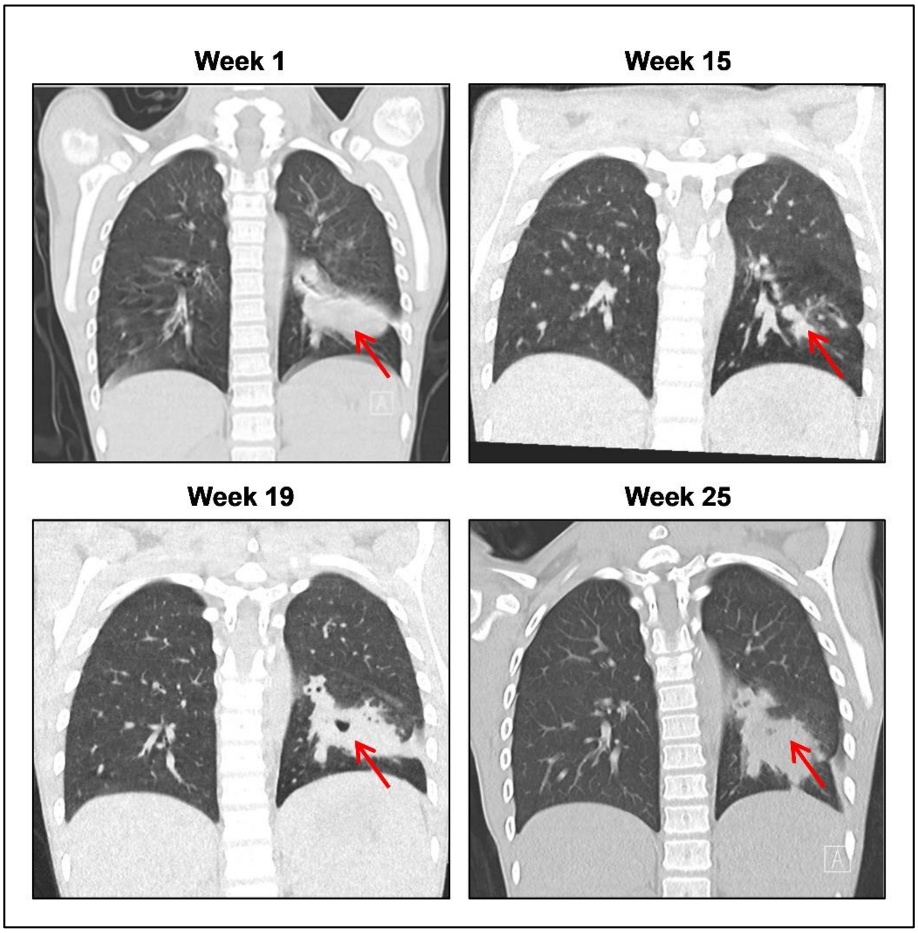

Supplementary Figure S1. Coronal views of chest CT at weeks 1, 15, 19, and 25. The red arrows point to the lung lesions.

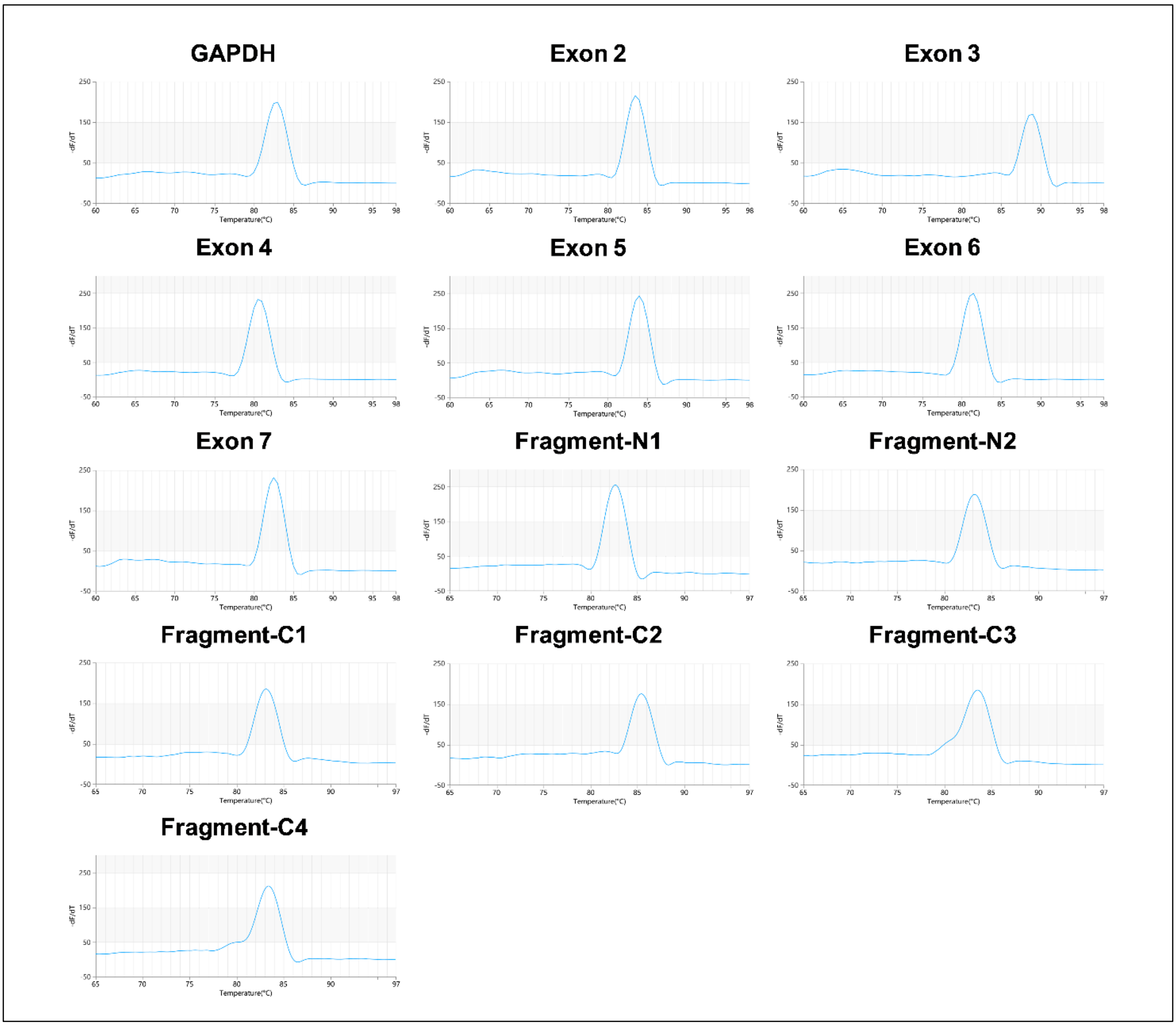

Supplementary Figure S2. Specificity of quantitative real-time PCR amplification. All melting curves were single peaks, indicating that the primers had good specificity and the experimental results were reliable.
